# *MycN* Is Critical for the Maintenance of Human Embryonic Stem Cell-Derived Neural Crest Stem Cells

**DOI:** 10.1371/journal.pone.0148062

**Published:** 2016-01-27

**Authors:** Jie Ting Zhang, Zhi Hui Weng, Kam Sze Tsang, Lai Ling Tsang, Hsiao Chang Chan, Xiao Hua Jiang

**Affiliations:** 1 Key Laboratory for Regenerative Medicine, Ministry of Education, Epithelial Cell Biology Research Center, School of Biomedical Sciences, The Chinese University of Hong Kong, Hong Kong SAR, PR China; 2 Department of Anatomical and Cellular Pathology, Faculty of Medicine, The Chinese University of Hong Kong, Hong Kong SAR, PR China; 3 The Chinese University of Hong Kong, Shenzhen Research Institute, Shenzhen, PR China; University of Montréal and Hôpital Maisonneuve-Rosemont, CANADA

## Abstract

The biologic studies of human neural crest stem cells (hNCSCs) are extremely challenging due to the limited source of hNCSCs as well as ethical and technical issues surrounding isolation of early human embryonic tissues. On the other hand, vast majority of studies on *MycN* have been conducted in human tumor cells, thus, the role of *MycN* in normal human neural crest development is completely unknown. In the present study, we determined the role of *MycN* in hNCSCs isolated from *in vitro*-differentiating human embryonic stem cells (hESCs). For the first time, we show that suppression of *MycN* in hNCSCs inhibits cell growth and cell cycle progression. Knockdown of *MycN* in hNCSCs increases the expression of *Cdkn1a*, *Cdkn2a* and *Cdkn2b*, which encodes the cyclin-dependent kinases p21^CIP1^, p16 ^INK4a^ and p15^INK4b^. In addition, *MycN* is involved in the regulation of human sympathetic neurogenesis, as knockdown of *MycN* enhances the expression of key transcription factors involved in sympathetic neuron differentiation, including *Phox2a*, *Phox2b*, *Mash1*, *Hand2* and *Gata3*. We propose that unlimited source of hNCSCs provides an invaluable platform for the studies of human neural crest development and diseases.

## Introduction

The neural crest originates in the ectodermal layer of developing vertebrate embryos. Through a series of highly orchestrated molecular signals, neuro-ectodermal cells are induced to first create neural crest stem cells (NCSCs). Subsequently, NCSCs respond to a variety of differentiation and migration signals to produce terminally differentiated neural crest (NC) derivatives including sympathetic and parasympathetic ganglia, adrenal medulla, connective tissues, and an array of different cell types [1]. At each step of this process, stage-and cell type-specific gene expression ensures successful tissue development. Disruption of this gene expression cascade at any point leads to aberrant proliferation, apoptosis, and/or differentiation.

*MycN* is one member of the *Myc* proto-oncogene family that includes c-*Myc* and *MycL*. The gene was first identified in neuroblastoma cell lines as amplified oncogene with homology to viral *Myc* [2]. *MycN* is primarily expressed in the early stage of embryonic development[3, 4], in contrasting to the expression of *c-Myc* throughout an animal’s life[5]. Strikingly, mouse embryos deficient in *MycN* die around E11.5 and display overwhelming hypoplasia in many organs and tissues including central and peripheral nervous system [4, 6]. In most organs and tissues, *MycN* is normally expressed in progenitor populations. However, as the cells commit to more differentiated states in concomitant with the progressive maturation of organs and tissues, *MycN* expression is turned off. This expression pattern implies that *MycN* and the broad transcriptional program it directs function in a general manner to maintain cells in a proliferative and undifferentiated state [3]. In agreement with the pro-proliferative role of *MycN*, disruption of *MycN* in neural precursor cells severely impairs brain growth, particularly that of the cerebellum in both mouse and human [5, 7, 8]. Furthermore, *MycN*-specific domains are conserved among the animal species, indicating *MycN* regulates transcription of a particular group of genes that are involved in the development process[9].

In the developing chicken and mouse embryos, many of mesectodermal tissues derived from the neural crest express *MycN* at a high level [5, 9]. *MycN* deficient mice exhibit dramatic reduction in central and peripheral ganglion sizes, indicating limited neural crest cells colonizing in the ganglia. In addition, *MycN* has been demonstrated to play critical roles in regulating neural crest migration and differentiation as illustrated in mouse and chicken embryos [10, 11]. In human, while large amount of studies on *MycN* have been conducted in human tumor cells, the role of *MycN* in human neural crest development is completely unknown largely due to the lack of appropriate cell model. Although human neural crest cells have been isolated from human adult tissues, they are exceedingly rare. On the other hand, induction and differentiation of embryonic neural crest occurs within a few weeks of fertilization [12, 13]—long before most women realize that they are pregnant. Thus, insights into human neural crest development will be most readily achievable using neural crest-directed differentiation of hESCs. In the present study, we determined the role of *MycN* in human NCSCs derived from human embryonic stem cells (hESCs). For the first time, we showed that suppression of *MycN* in hNCSCs inhibited cell growth and cell cycle progression via induction of *Cdkn1a*, *Cdkn2a* and *Cdkn2b*. In addition, *MycN* is involved in the differentiation of human sympathetic neurons.

## Materials and Methods

### Cell Culture

Human embryonic stem cells (hESC) H9 (WA-09, WiCell Research Institute, Madison, WI, USA) was cultured on Mitomycin C-treated mouse embryo fibroblast (EmbryoMax^®^ Primary Mouse Embryo Fibroblasts, Strain CF1, Merck Millipore, Massachusetts, USA) in hESC culture media as previously described[14]. The undifferentiated phenotype of hESCs has been validated by immunofluorescent and FACS analyses (S1 Fig). For neural crest differentiation, hESC colonies were treated with collagenase IV, mechanically sectioned into clumps and transferred into PA6 (Cell Bank, RIKEN BioResource, Ibaraki, Japan)-coated dishes at densities of up to 500 colonies per 3 cm dish. Media was then changed to NC induction media. On day 6, 1X N2 supplement (Life Technologies, Carlsbad, CA) was added to the induction media and replaced every 2 days thereafter as previously described [14, 15].

### FACS Analysis and Purification

H9 colonies were dissociated by Accumax (Chemicon, Temecula, CA) and blocked with anti-human Fc- receptor (Miltenyi Biotec, Bergisch Gladbach, Germany). Following Fc blocking, cells were incubated with the phycoerythrin (PE)-conjugated p75 antibody (557196, BD Pharmingen, CA) for 20 min at 4°C. For HNK-1 and p75 double staining, the cells were firstly stained with HNK-1-FITC (322306, Biolegend, CA), then followed by conjugated anti-p75-PE. Compensation for FITC and PE was performed using compensation beads (BD Pharmingen, San Jose, CA). Positive and negative gates were determined using IgG stained and unstained controls.p75^+^or HNK-1^+^/p75^+^hNCSC cells were routinely maintained in self-renewal medium [14]on 6-well ultra-low attachment plates (Corning, Lowell, MA) at a density of 5×10^3^ cells/ml.

### *MycN* knockdown in hNCSCs

To knockdown *MycN* expression in hNCSCs, hNCSCs were plated at a cell density of 4×10^3^ cells/cm^2^ in self-renewal media [14]on 6-well plates that were pre-coated with 15 μg/ml Polyornithine, 1 μg/ml laminin and 10 μg/ml fibronectin for 24 hours. Freshly isolated hNCSCs were transduced with concentrated pGLVH1/GFP-MycN shRNA virus (Shanghai GenePharma., Ltd, Shanghai, China) at a multiplicity of infection of 10. Transduced cells were selected in puromycin (2 μg/ml) for two weeks.

### Reverse Transcription–Polymerase Chain Reaction (RT-PCR) analysis

Total RNA extraction was performed using the TRIzol reagent (Life Technologies, Rockville, MD), according to the manufacturer's instructions. Amplification of transcripts was performed using 3–5μg/μl of total RNA. The reverse transcriptase-polymerase chain reaction (RT-PCR) was performed using moloney murine leukemia virus reverse transcriptase (MMLV) and oligo-d(T)15 primer. Specific primers were purchased from Life technologies and listed in S1 Table. The cDNA samples were 2.5 X diluted by H_2_O as template for quantitative PCR assay. QPCR reactions were carried out in triplicate on 96-well plate using an Applied Biosystems 7500Fast Real-Time PCR System. To calculate the relative transcriptional expression, the Ct values of interested genes were normalized by average Ct values of *gapdh* as ΔCt, then the ΔCt of *MycN*kd cells were normalized by ΔCt of control cells to get the ΔΔCt. The relative transcriptional expression of interested genes was indicated with2^(-ΔΔCt)^.

### Fluorescent Immunocytochemistry

Cells were grown on Lab-Tek chamber slides (Nalge Nunc International, Rochester, NY). For immunofluorescent staining, cells were fixed in 4% paraformaldehyde and blocked with 1% bovine serum albumin, and incubated overnight with primary antibodies at 4°C. The secondary antibodies at a dilution of 1:500 were loaded on cells for 1 hour. Following three washing with PBS, the slides were mounted and visualized with fluorescence microscopy. The following antibodies were used at the indicated dilutions: p75 (1: 100; Millipore, AB1554), HNK-1 (1:100; Millipore, CBL519). The staining omitting the primary antibodies was used as negative control.

### Cell Growth, Cell Cycle Analysis and Neural Crest Differentiation

Cell growth rate was measured with an MTT proliferation assay. The Cell Titer 96 Aqueous One Solution Cell Proliferation Assay (Promega) was conducted according to the manufacturer’s instructions. Cell cycle analysis was performed by flow cytometric analysis of ethanol-fixed cells using propidium iodide staining and analyzed on BD FACSAria. For non-specific neural crest differentiation of hNCSCs, self-renewal media was changed to DMEM/F12 (1:1) supplemented with N2, B27 and 5% Hyclone^®^ fetal bovine serum (FBS) (Thermo scientific) and incubated for consecutive 21 days.

### Western Blot

Cells were collected and washed once using PBS. RIPA buffer (150mM NaCl, 50mMTris-Cl, 1% NP-40, 0.5% DOC, 0.1% SDS, 1:100 PMSF, and 1:200 PImix) was used to lyse cells for 60 min at 4°C. Supernatant was collected as total protein after centrifugation at 12,000 rpm for 30 min. Equal amounts of protein were separated by SDS-PAGE and detected for target proteins. The protein bands were visualized by the enhanced chemo-luminescence (ECL) assay (Amersham) following manufacturer's instructions and scanned by densitometer. Antibodies used are c-Myc (Cell Signaling Technology, CSC5605P), MycN (Thermo Fisher, #MA1-16638), Cyclin D1 (Santa Cruz, sc-8396), p15 (Santa Cruz, sc-612), p16 (BD, BD550843), p21 (Santa Cruz, sc-6246).

### Statistical Analysis

Values are reported as means ± SD if not indicated otherwise. Unpaired t test with Welch's correction was used for comparing the gene expression, cell growth and cell cycle differences between control and *MycN* knockdown cells.

## Results

### Isolation of hNCSCs from Neural Crest Differentiating hESCs

**Fig 1:**
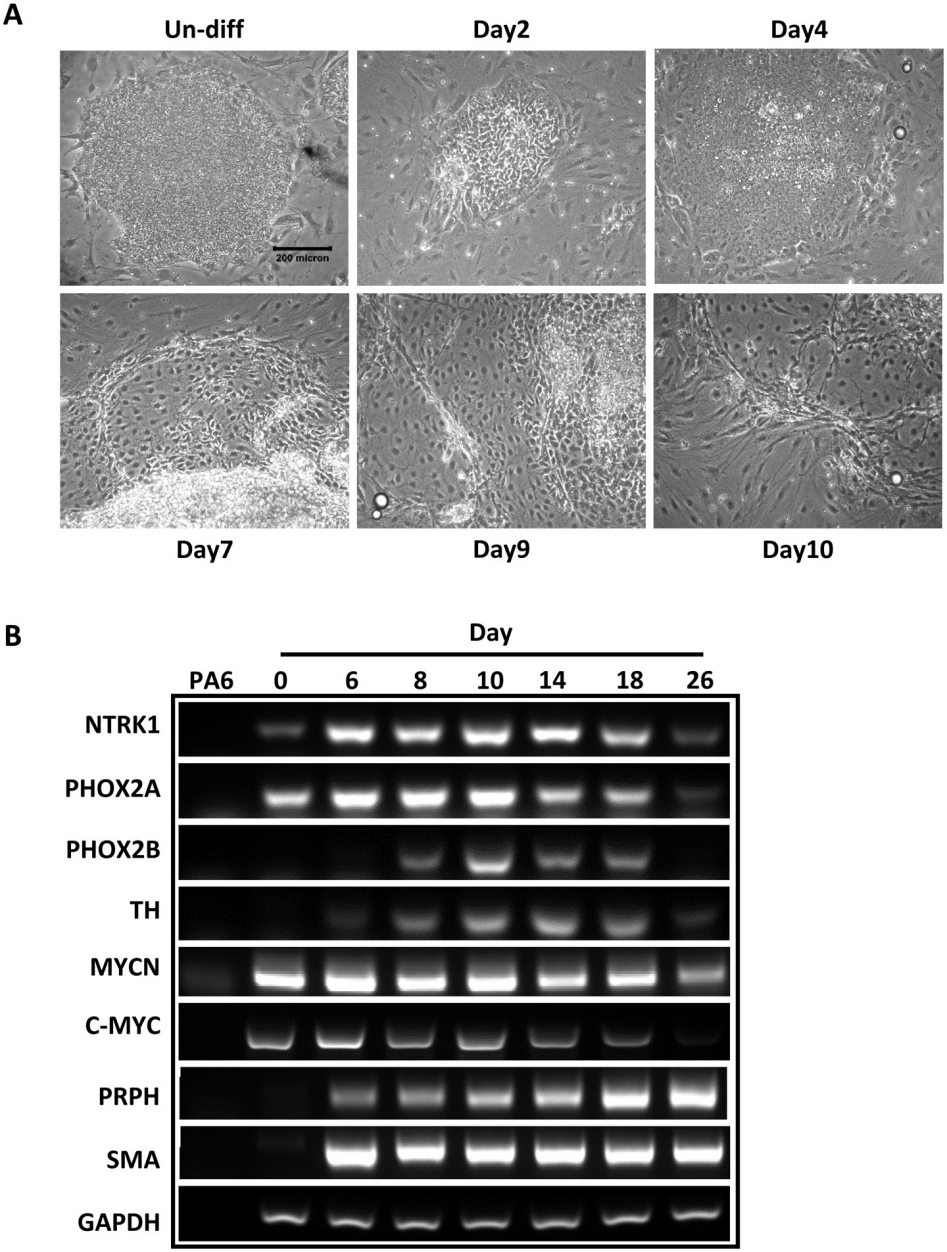
Induction of neural crest cells from hESCs. For neural crest induction, H9 cells were plated at 5-10x10^2^/cm^2^ on a confluent layer of PA6 cells in 6 well plates or chamber slides in induction medium as described in *Materials and Methods*. **(A):**Phase contrast photographs of neural crest differentiation of H9 induced by PA6, scale bar = 200μm;**(B):**Dynamic expression of sympathetic neuronal markers, *c-Myc* and *MycN* in NC differentiating cells from hESCs as determined by PCR. The image is the representative of three independent experiments.

**Fig 2:**
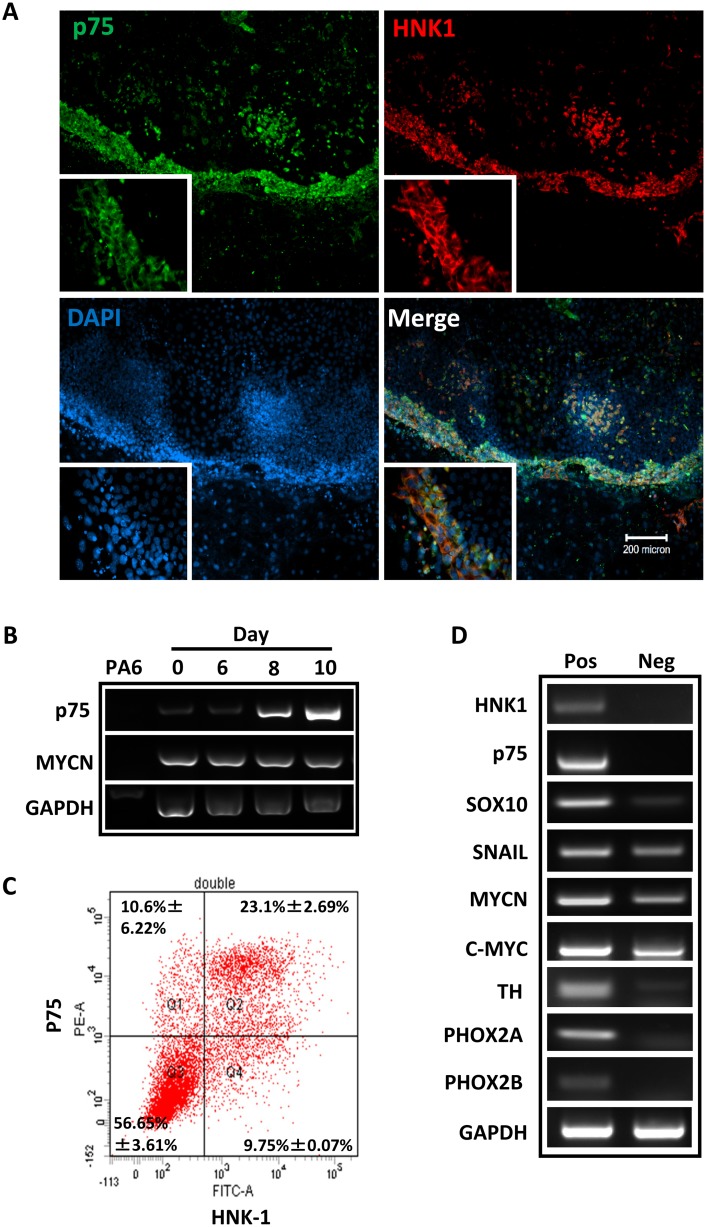
Induction and isolation of hNCSCs from hESCs. **(A):** A colony of H9 hESCs double stained for p75 (green) and HNK-1 (red) after one week of culture on PA6. Note that the expression of p75 and HNK-1 is outside or at the edge of the hESC colony, scale bar = 200μm; **(B):** Reverse transcription-polymerase chain reaction analysis (RT-PCR) of *p75* expression, note that the expression of *p75* is gradually increased up to day 10; **(C):** Analysis of H9-derived p75^+^ and HNK1^+^ cells by FACS analysis. The dot plots are representative of three independent experiments; **(D):** Different gene expression profiles are displayed in p75^+^ and p75^-^ populations as assessed by RT-PCR. Note that p75^+^ cells express markers of NCSC and sympathetic neurons. The image is the representative of three independent experiments.

Using a well-established protocol, neural crest differentiation of hESCs was initiated by stromal cell-derived inducing activity (SDIA) as previously described[14]. We observed prominent morphology changes started on day 7 of induction. Subsequent changes indicating neural induction, such as fiber bundles formed by processes emanating from the colonies, appeared frequently on day 10 after co-culture (Fig 1A). Since *MycN* has been implicated in the regulation of sympathetic neurogenesis [16, 17], we used RT-PCR to examine the expression of sympathetic neuronal markers, such as neurotrophic receptor tyrosine kinase 1 (*Ntrk1*), tyrosine hydroxylase (*TH*), *Phox 2a*and *Phox2b*, over the course of SDIA exposure (Fig 1B). Semi-quantitative evaluation of the expression of these genes showed a temporal induction started at around one week, and followed by a down-regulation after two weeks. In contrast, the expression of terminal neural crest differentiation markers, such as *peripherin* and *SMA*, was induced at around one week and maintained high afterward. Of note, while SDIA induction did not change the expression level of *MycN* and *c-Myc* during the early differentiation, the expression of *MycN* and *c-Myc* dramatically decreased after three weeks of differentiation (Fig 1B and S2 Fig). To further identify populations of hNCSCs within differentiating hESC cultures, we evaluated the expression of NCSC markers by immunostaining and FACS analysis. By one week of co-culture, expression of p75 and Hnk-1, markers for NCSCs[18, 19], was upregulated and could be observed predominantly localized at the periphery of H9 colonies (Fig 2A & 2B). In addition, our FACS analysis demonstrated that at day 8–9 of SDIA induction, more than 30% of cells were p75 positive. Similar temporal analysis of Hnk1 expression by FACS showed that most hESC–derived p75^+^ cells also expressed Hnk1 (Fig 2C). Since p75 has been widely used to characterize and isolate NCSCs [18–20], in the following study, we used p75 to isolate hNCSCs at day 8 of SDIA induction. We sorted p75^+^ and p75^-^ cells by FACS at day 8 of SDIA induction and evaluated the expression of neural crest and sympathetic neuronal markers by RT-PCR (Fig 2D). As shown in Fig 2D, transcripts highly enriched were markers for NCSCs (*p75*, *Sox10*, *Hnk1*, *Snail*) and sympathetic neurons (*TH*, *Phox2a*, *Phox2b*) in the p75^+^ fraction but not p75^-^ fraction, indicating markers of neural crest identity are enriched inp75^+^ cell fractions. In addition, we compared the expression level of *MycN* and *c-Myc* in p75^+^ and p75^-^ populations. Our results showed that while the expression level of *MycN* was significantly higher than *c-Myc* in the p75^+^ population, both *MycN* and *c-Myc* were more enriched in the p75^+^ fraction (Fig 2D and S3 Fig).

### *MycN* Is Critical for Cell Growth and Cell Cycle Progression in hNCSCs

**Fig 3:**
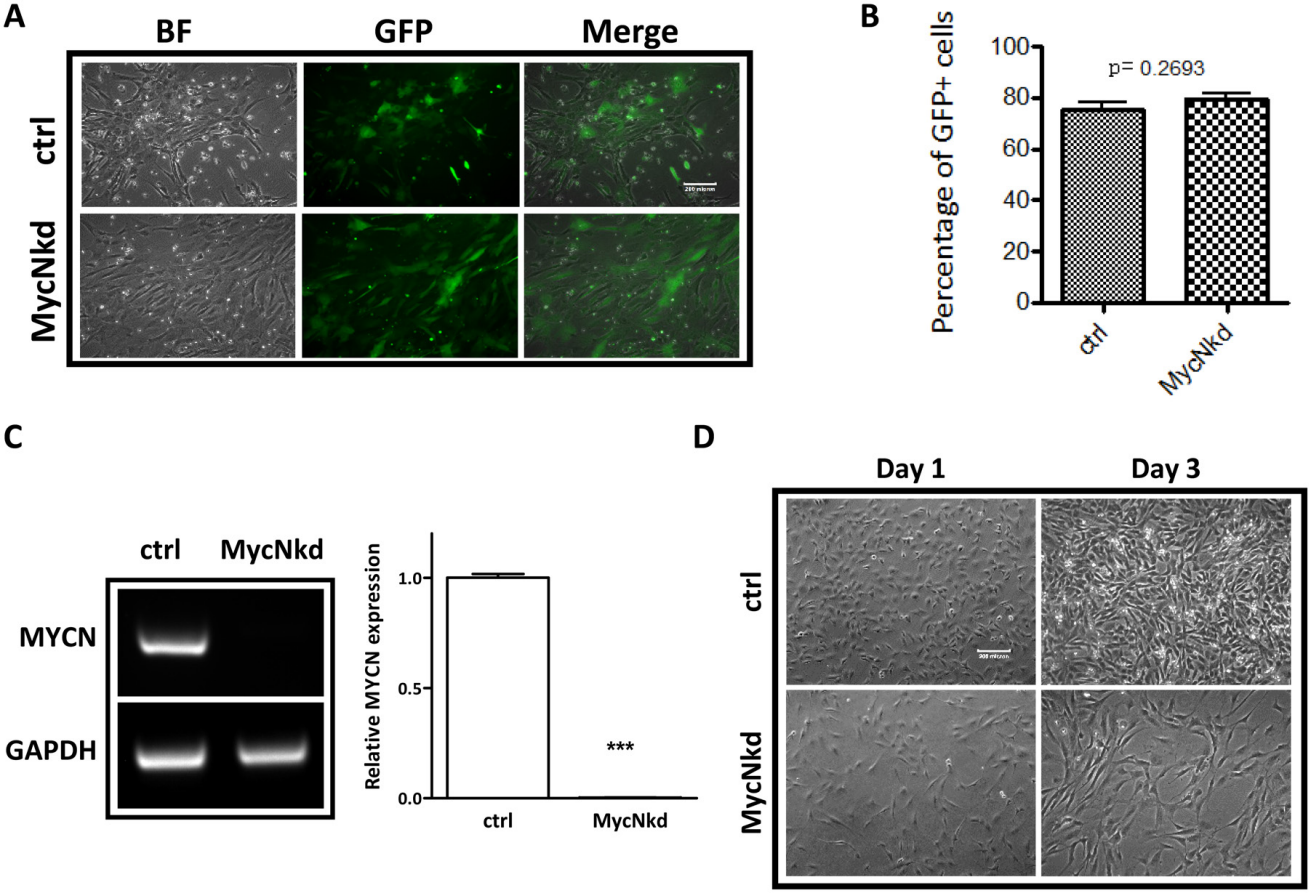
*MycN* knockdown inhibits cell growth in hNCSCs. FACS-sorted p75^+^ hNCSCs were plated at a cell density of 4×10^3^ cells/cm^2^ in self-renewal media on 6-well plates that were pre-coated with 15 μg/ml polyornithine, 1 μg/ml laminin and 10 μg/ml fibronectin for 24 hours. Freshly isolated hNCSCs were transduced with concentrated pGLVH1/GFP-*MycN*shRNA virus and selected in puromycin (2 μg/ml) for two weeks, as described in Materials and Methods.**(A):** The GFP expression in control and *MycN*-transduced hNCSCs; **(B):** FACS analysis shows the similar transduction efficiency in control and *MycN*-transduced hNCSCs; **(C):** RT-PCR analysis showed the expression of *MycN* in control and *MycN*kd hNCSCs after 2 weeks of antibiotics selection. Image shown is the representative of three independent experiments; **(D):** Freshly sorted cells were plated onto 15 μg/ml polyornithine, 1 μg/ml laminin and 10 μg/ml fibronectin coated plates at a concentration of 10x10^3^ cells/well and grown in NC media. Within two days to adherent plate, *MycN* knocked down cells initiated a change from small, NCSC-like to a larger shape.

**Fig 4:**
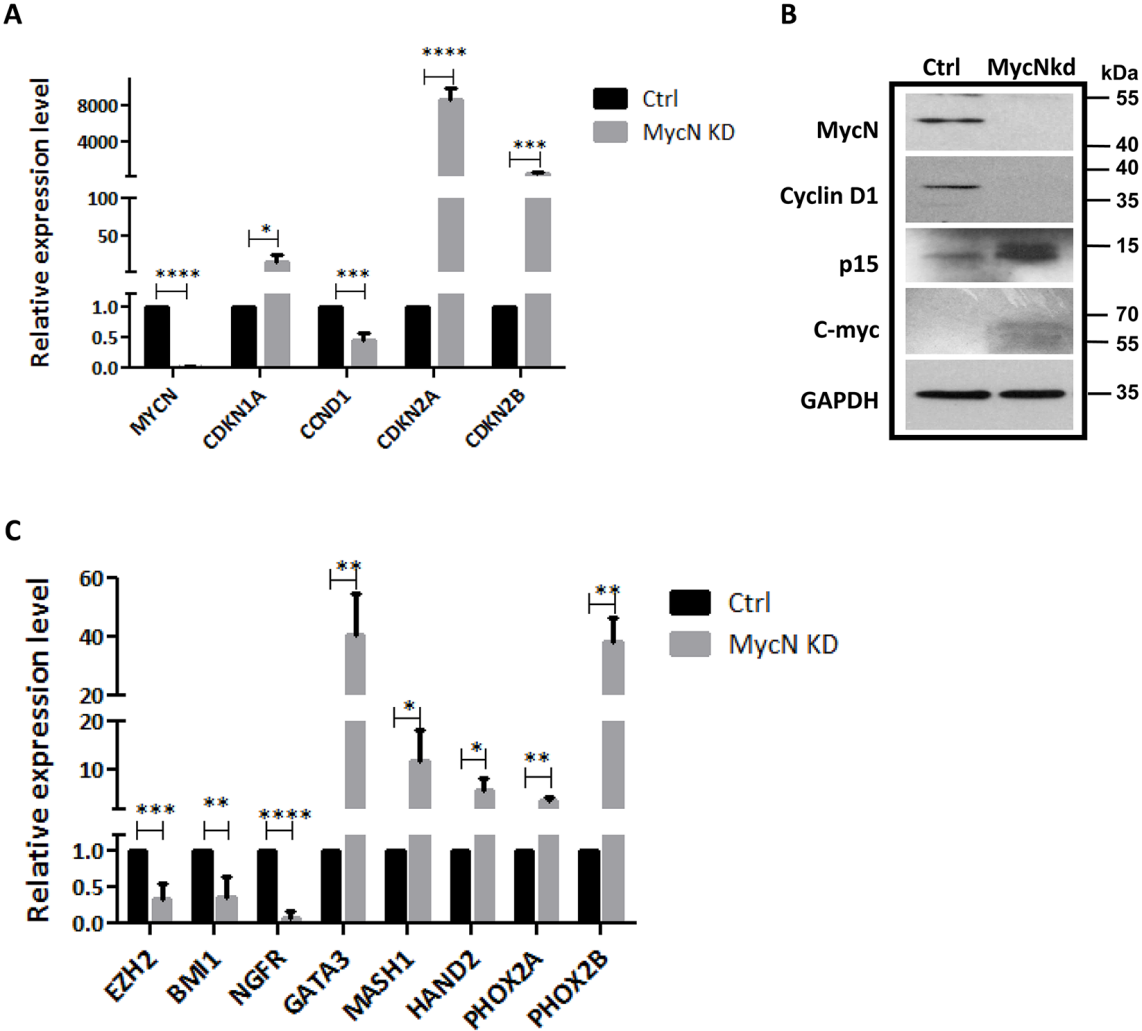
*MycN* knockdown affects the expression of cell cycleand autonomic neuron development-related genes. FACS-sorted p75^+^ hNCSCs were transduced with concentrated pGLVH1/GFP-MYCN shRNA virus and selected in puromycin for two weeks.**(A):** Real-time PCR analysis showing the expression of cell cycle-related genes was altered by *MycN* suppression, *p<0.05, ***p<0.001; ****p<0.0001; quantitative data was acquired from three independent experiments;**(B):** Western blot shows knockdown of *MycN* increases the expression of p15, whereas decreases the expression of Cyclin D1; **(C)** Real-time PCR analysis showing the expression of polycomb genes and autonomic neuronal markers was altered by *MycN* suppression, *p<0.05, **p<0.01;***p<0.001; ****p<0.0001, quantitative data were acquired from three independent experiments.

**Fig 5:**
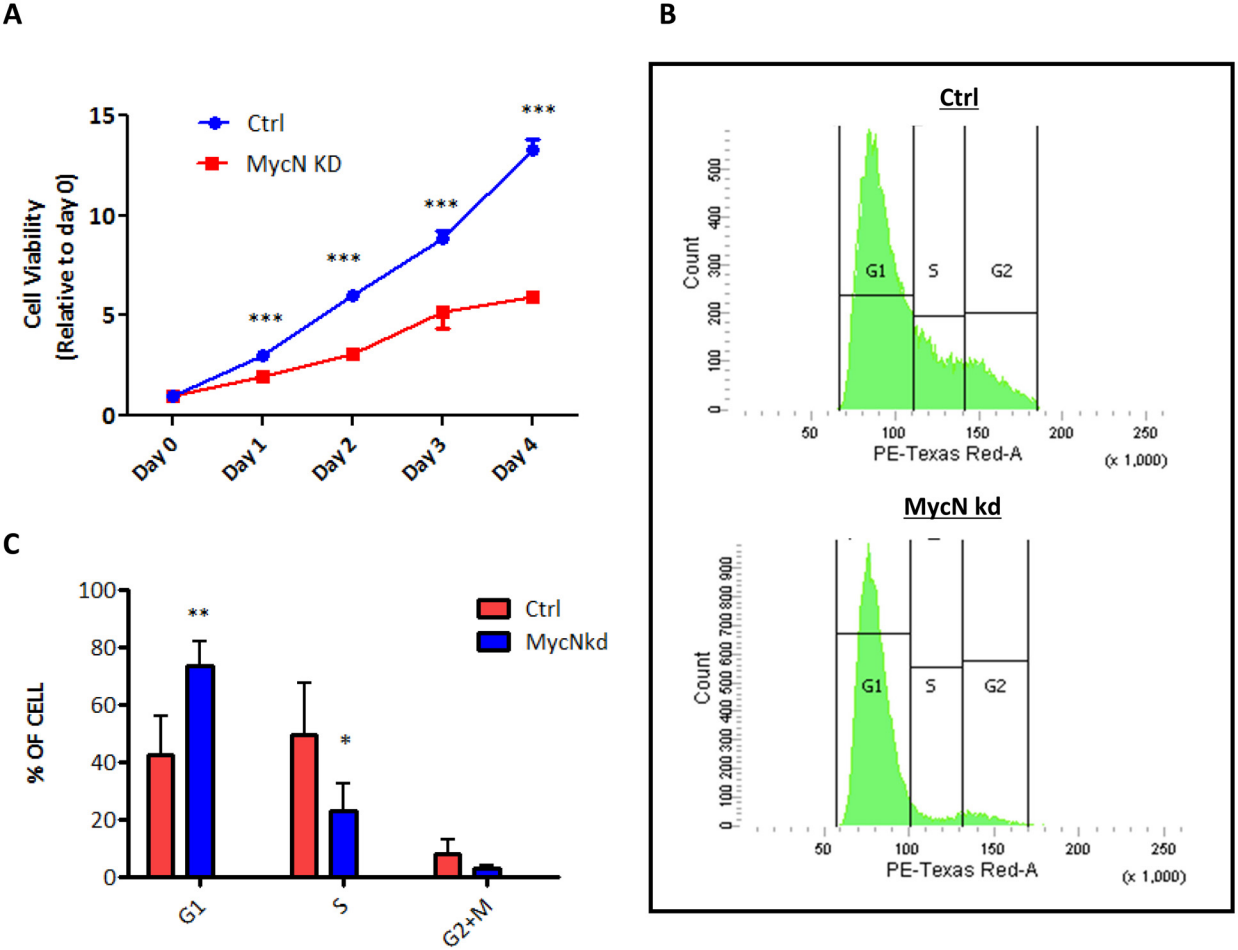
*MycN* knockdown suppresses cell cycle progression in hNCSCs. FACS-sorted p75^+^ hNCSCs were transduced with concentrated pGLVH1/GFP-MYCN shRNA virus and selected in puromycin for two weeks.**(A):** Cell growth of control and *MycN*kd hNCSCs was detected by MTT assay, cell growth is presented as percentage increase: Absorbance _dn_–absorbance _d0_ / absorbance _d0_and error bars representing 95% confidence intervals are shown. Data are from three independent experiments, ***p<0.001; **(B):** Cell cycle was assessed in adherent control and *MycN*kd hNCSCs 2weeks after lentiviral transduction; **(C)**: Flow cytometric of DNA content showed *MycN* kd hNCSCs was arrested in G0/G1, *p<0.05, **p<0.01.

To understand whether or not *MycN* has any effect on hNCSCs, freshly isolated hNCSCs were transduced with control-shRNA or *MycN*-shRNA lentiviral vectors and then evaluated for cell morphology and cell growth. After 2 weeks of selection, we achieved high transduction efficiency as demonstrated by GFP expression in both control and *MycN* knockdown hNCSCs (Fig 3A & 3B). Our RT-PCR and western blot analyses confirmed dramatic suppression of *MycN* expression in *MycN*shRNA (*MycN*kd) treated-hNCSCs compared to control (Ctrl, Figs 3C & 4B). Of note, while the expression of *c-Myc* was much lower than *MycN* in hNCSCs (S3B Fig), knockdown of *MycN* mildly increased the expression of *c-Myc* at protein level (S4A Fig and Fig 4B). Interestingly, substantial differences in cell morphology and cell proliferation were observed between Ctrl and *MycN*kd hNCSCs. While control cells were generally smaller and more NCSC-like in appearance, *MycN*kd cells displayed large fibroblastic morphology (Fig 3D). Importantly, although started with the same number of cells, there was much less cells in *MycN*kd group after 1 day culture (Fig 3D). Since we did not observe increased cell death in *MycN*kd population, it is very likely that the difference in cell numbers is attributed to proliferative capacity. Indeed, although control cells could be passaged and maintained their self-renewal capacity in vitro for up to 2 months, *MycN*kd hNCSCs ceased to proliferate after 4 weeks in culture. We then studied the possible role of *MycN* in hNCSC proliferation and cell cycle progression. MTT assay was used as a measure of the relative inhibition of cell proliferation in *MycN*kd compared to Ctrl hNCSCs. As depicted in Fig 4A, depletion of *MycN* decreased cell proliferation in a time-dependent manner. Suppression of *MycN* dramatically inhibited cell proliferation by about 50% after 4 days of incubation. In addition, our cell cycle analysis showed that *MycN* knockdown arrested cell cycle in the G0/G1 phase(Fig 5B & 5C). To further understand how *MycN* regulates cell cycle progression, we evaluated the effect of *MycN* knockdown on well-established cell cycle-related genes. Our results showed that after 1 week of *MycN* knockdown, there was significant increase in the expression of well-recognized tumor suppressors that induce G1 cell cycle arrest, such as *Cdkn1a*, *Cdkn2a* and *Cdkn2b* (Fig 4A). In addition, the expression of *Cyclin D1*, whose activity is required for cell cycle G1/S transition, was significantly downregulated in *MycN*kd cells compared to control (Fig 4A). Our western blot result confirmed the regulatory effect of *MycN* knockdown on p15 and Cyclin D1 (Fig 4B and S5 Fig). Taken together, our results showed that *MycN* is critical for hNCSC proliferation and cell cycle progression.

### Suppression of *MycN* Promotes Sympathetic Neurogenesis

Dysregulated differentiation of sympathetic progenitor cells plays a key role in neuroblastoma pathogenesis, which is well exemplified in a mouse model of neuroblastoma that arises from the developing sympathetic ganglia or adrenal medulla [21]. However, the question of whether *MycN* is implicated in the differentiation of human sympathetic neurons is unknown. Thus, we decided to examine the expression of key regulators of sympathetic neurogenesis in *MycN* knockdown and control hESC-derived hNCSCs by real-time PCR. Paired-like homeobox transcription factors *Phox2a* and *Phox2b* are essential for the specification of the autonomic nervous system. Thus, we first determined the expression of the two key transcription factors involved in the sympathetic neurogenesis and found that *MycN* knockdown significantly upregulated the expression of *Phox2a* and *Phox2b* (Fig 4C). Next, the expression of *Phox2* downstream targets that are involved in sympathoadrenal differentiation was examined in control and *MycN* knockdown hNCSCs. Our results showed that suppression of *MycN* significantly upregulated the expression of*Mash1*, *Hand2* and *Gata3 as well* (Fig 4C). Altogether, these results suggest that *MycN* regulates NCSC-directed sympathetic neurogenesis. Recent studies have indicated that polycomb genes, such as *Bmi-1* and *Ezh2*, control the transcriptional activation of neurogenesis genes by epigenetic machinery. *MycN* regulates the expression of these polycomb genes and contributes to the pathogenesis of neuroblastoma [15, 22, 23]. Thus, we determined whether manipulation of *MycN* in hNCSCs had any effects on the expression of *Ezh2* and *Bmi-1* in our system. Interestingly, knockdown of *MycN* significantly downregulated the expression of *Ezh2* and *Bmi-1* in hNCSCs (Fig 4C). To address whether suppression of *MycN* impedes NCSC differentiation into multi-lineage neural crest derivatives *in vitro*, control and *MycN*kd hNCSCs were plated on poly-D-lysine/fibronectin coated plates for two weeks in the presence of defined medium plus 5%FBS. Our results showed that knockdown of *MycN* in hNCSCs did not change the expression of *SMA* and *Tuj1*, but mildly downregulated the expression of *MAP2*, indicating the role of *MycN* in lineage specification (S4B Fig).

## Discussion

Numerous previous studies have documented the destructive effects of *MycN* loss on early mouse development [5, 24–26]. However, the exact function of *MycN* in neural crest stem/progenitor cells remains largely unknown. Interestingly, previous studies in model systems indicated the critical role of *MycN* in neural crest cell differentiation and migration [11]. It was reported that overexpression of *MycN* in avian neural crest cells promoted cell migration and increased generation of neurons [10]. Similarly, overexpression of tyrosine hydroxylase (TH) driven-*MycN* in the murine neural crest in transgenic mice pushed progenitors towards a neuronal fate [16]. These results indicate that the functional role of *MycN* in NCSCs may not be necessarily identical to that in neural progenitor cells. Thus, NCSCs in vitro culture model will be useful in deciphering whether the acute response of NCSCs to *MycN* deficiency is a failure of proliferation or differentiation. In the current study, we have shown that knockdown of *MycN* inhibits cell proliferation in hNCSCs. Compared to control cells, which could be maintained for 2–3 months, the *MycN* depleted hNCSCs could only be passaged for 2–3 weeks. Following cell cycle analysis showed that downregulation of *MycN* dramatically impaired cell cycle progression and arrested hNCSCs at G0/G1 phase (Fig 5). These results provide strong evidence that *MycN* is critically important for the sustained proliferation of hNCSCs. One interesting finding from this experiment is that while the expression level of *c-Myc* does not change after *MycN* knockdown (S4A Fig), *c-Myc* cannot compensate the effect of *MycN* on hNCSC proliferation, indicating *MycN* may dominate the regulatory role of cell growth regulation in hNCSCs. This notion is further supported by the finding that *MycN* is highly expressed in p75^+^ population (Fig 2D and S3B Fig). Of surprise, we did not observe significant increase in apoptosis in *MycN* knockdown hNCSCs. This finding is in consistence with the *MycN* knockout mice study showing that no obvious apoptosis is detected in the spinal and dorsal root ganglia[5]. Altogether, it is plausible that *MycN* regulates NCSCs proliferation, rather than apoptosis.

The molecular mechanism for the control of sympathetic neurogenesis has been largely elucidated. In response to bone morphogenetic proteins (BMPs) produced and secreted by the dorsal aorta, sympathetic neural crest cells express the pro-neural genes *Mash1* and *Phox2b* and adopt a neuronal fate, which in turn promote further neuronal differentiation by regulating the expression of transcription factors *Hand2*and *Gata3*[27, 28]. These transcription factors collaborate in a complex regulatory network to specify the noradrenergic phenotype of sympathetic neurons [17]. Intriguingly, numerous studies have reported that oncogenes such as *MycN* and *ALK* dysregulate the embedded sympathetic neurogenesis program, which facilitates the initiation of tumorigenesis [16, 29]. *Phox2b* is a “master regulator” of sympathetic neuronal development and mainly expressed in sympathetic neural progenitors [17, 28, 30]. *Phox2b* also has growth inhibitory effects, as its overexpression promotes cell cycle exit and inhibits the proliferation of cultured sympathetic neurons [31, 32]. In the hNCSCs, we have found that suppression of *MycN* dramatically upregulates the expression of *Phox2b*, and its downstream target genes *Hand2*, *Mash1* and *Gata3*, indicating suppression of *MycN* expression in hNCSCs promotes autonomic neuron development. The regulatory effects of *MycN* on *Phox2b* and its downstream targets indicate *MycN* controls development progression of autonomic neurogenesis at sympathetic progenitor stage, while the exact mechanisms by which these master regulators of sympathetic neurogenesis are regulated need further investigation.

Although the underlying mechanisms responsible for the many defects caused by loss of *MycN* remain a mystery, *Myc* family proteins participate in the repression of several key genes that regulate cell cycle. Two critical targets of *Myc* regulation are *Cdkn1a* and *Cdkn2b*, which encode the cyclin-dependent kinases p21^CIP1^ and p15^INK4b^ [33–35]. Consistently, we have illustrated that *MycN* knockdown in human NCSCs increased the expression of *Cdkn1a*, *Cdkn2a* and *Cdkn2b* (Fig 4A & 4B), emphasizing the critical role of these tumor suppressors in stem cell growth and cell cycle progression. On the other hand, accumulating evidence has shown that *MycN* regulates the expression of polycomb genes, which are critical for the maintenance of stem cell self-renewal[22]. Polycomb genes, such as *Bmi1*and *Ezh2*, block the transcriptional activation of differentiation genes by epigenetic suppression. Intriguingly, although binding of *MycN* was generally associated with active chromatin marks, transcriptional repression chromatin remodeling factors such as *Bmi-1*, *Ezh2* and *Suz12*, were detected around the E-box sequence in the presence of *MycN*. This ‘bivalent’ configuration is typical of repressed, developmentally regulated genes, which are poised to be activated by physiological stimuli during differentiation [36]. In our study, we found that knockdown of *MycN* downregulated the expression of *Bmi1* and *Ezh2* in hNCSCs, indicating polycomb genes might be involved in the effects of *MycN* on hNCSC differentiation.

To date, studies of neural crest development and biology have been almost exclusively done using model organisms. With the discovery of hESCs, there is no doubt that knowledge of human neural crest development will be profoundly enhanced by the unique opportunities provided by NC lineage-directed differentiation of hESCs. In our study, for the first time, we have demonstrated that *MycN* governs human neural crest stem cell growth and cell cycle progression. We propose that in vitro differentiation from hESCs can be reliably used to isolate large numbers of human NCSCs for biologic study of human neural crest development and diseases.

## Supporting Information

S1 Fig Immunofluorescent characterization and representative FACS analysisof H9 hESC confirms undifferentiated phenotype. **(A):** Adherent H9 cells were stained with Oct4, TRA-1-60, SSEA-4 and SSEA-1.**(B):** Adherent H9 cells were treated with collagenase, stained with PE conjugated antibodies against SSEA-4 and SSEA-1 for 30 minutes at 4°C and then analyzed by FACS Calibur (BD Biosciences).
(PDF)

S2 Fig Dynamic expression of *c-Myc* and *MycN* in neural crest differentiating hESCs. The expression of c-Myc and MycN was determined by western blot in differentiating hESCs in day0, 1week, 2 2weeks and 3 weeks.
(PDF)

S3 Fig The expression of *MycN* and *c-Myc* in p75^+^ and p75^-^ populations. **(A):** FACS analysis shows the p75^+^ population for sorting. **(B):** The expression of *MycN* and *c-Myc* in p75^+^ and p75^-^ populations determined by real-time PCR.
(PDF)

S4 Fig *MycN* knockdown had mild effect on neuronal differentiation. **(A):** Real time PCR analysis showed the knockdown of *MycN*did not affect the expression of *c-Myc*in hNCSCs; **(B):** Control and *MycN*kd cells were plated on poly-D-lysine- and fibronectin-coated coverslips in differentiation media for 2 weeks and then determined the expression of *SMA*, *Tuj1*, and *MAP2* by RT-PCR.
(PDF)

S5 Fig Original western blots showing knockdown of *MycN* increases the expression of p15, whereas decreases the expression of Cyclin D1. (PDF)

S1 Table Primer lists used in this study. (PDF)
