# Correction: *MycN* Is Critical for the Maintenance of Human Embryonic Stem Cell-Derived Neural Crest Stem Cells

**DOI:** 10.1371/journal.pone.0154068

**Published:** 2016-04-14

**Authors:** Jie Ting Zhang, Zhihui Weng, Kam Sze Tsang, Lai Ling Tsang, Hsiao Chang Chan, Xiao Hua Jiang

The second author’s name is spelled incorrectly. The correct name is: Zhihui Weng. The correct citation is: Zhang JT, Weng Z, Tsang KS, Tsang LL, Chan HC, Jiang XH (2016) *MycN* Is Critical for the Maintenance of Human Embryonic Stem Cell-Derived Neural Crest Stem Cells. PLoS ONE 11(1): e0148062. doi:10.1371/journal.pone.0148062

## References

[1] Zhang JT, Weng ZH, Tsang KS, Tsang LL, Chan HC, Jiang XH (2016) *MycN* Is Critical for the Maintenance of Human Embryonic Stem Cell-Derived Neural Crest Stem Cells. PLoS ONE 11(1): e0148062 doi:10.1371/journal.pone.0148062 26815535 10.1371/journal.pone.0148062 PMC4729679

