# Supplementary figures and images for: MycN Is Critical for the Maintenance of Human Embryonic Stem Cell-Derived Neural Crest Stem Cells
Source: PLoS One. 2016 Jan 27;11(1):e0148062. doi: 10.1371/journal.pone.0148062 (PMC4729679; doi:10.1371/journal.pone.0148062)

A

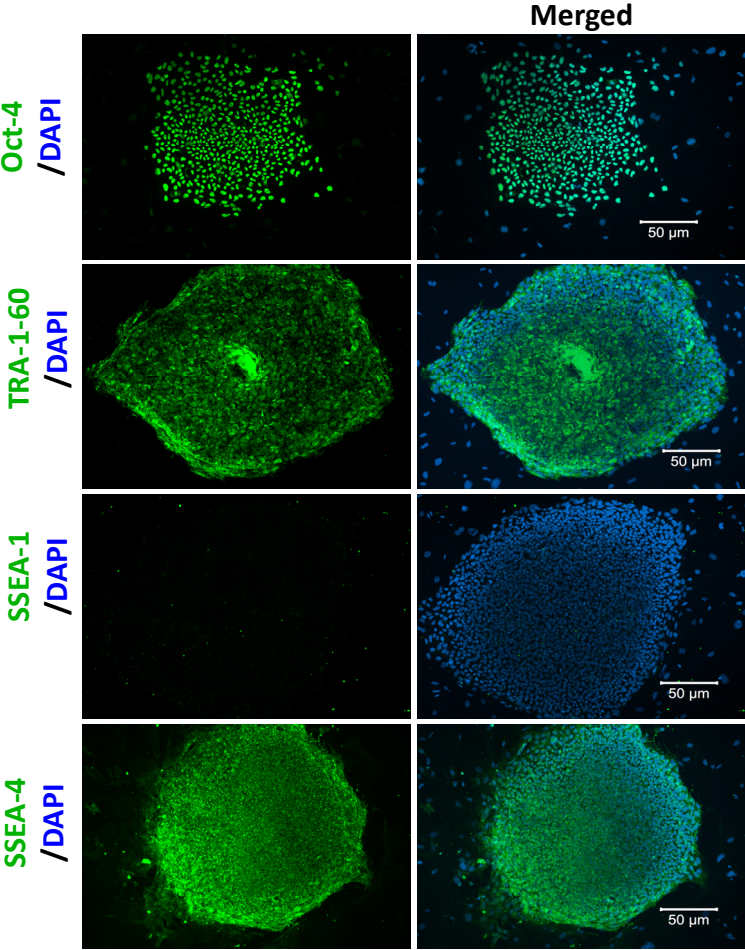

B

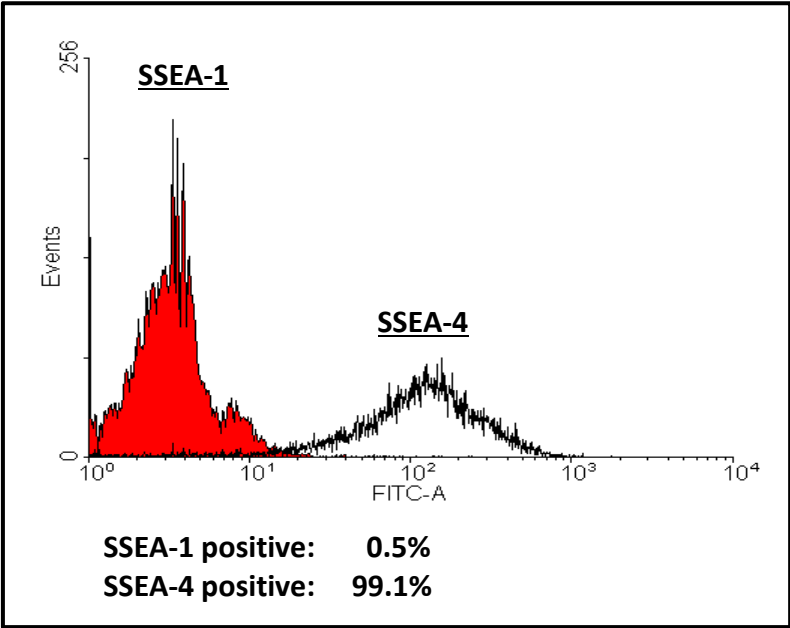

Supplement: S1 Fig — (A): Adherent H9 cells were stained with Oct4, TRA-1-60, SSEA-4 and SSEA-1.(B): Adherent H9 cells were treated with collagenase, stained with PE conjugated antibodies against SSEA-4 and SSEA-1 for 30 minutes at 4°C and then analyzed by FACS Calibur (BD Biosciences). (PDF) [file pone.0148062.s001.pdf]

**S2 Fig**

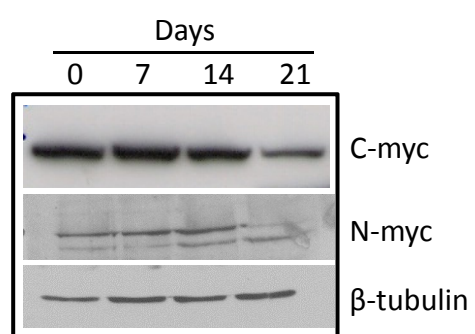

Supplement: S2 Fig — The expression of c-Myc and MycN was determined by western blot in differentiating hESCs in day0, 1week, 2 2weeks and 3 weeks. (PDF) [file pone.0148062.s002.pdf]

**A**

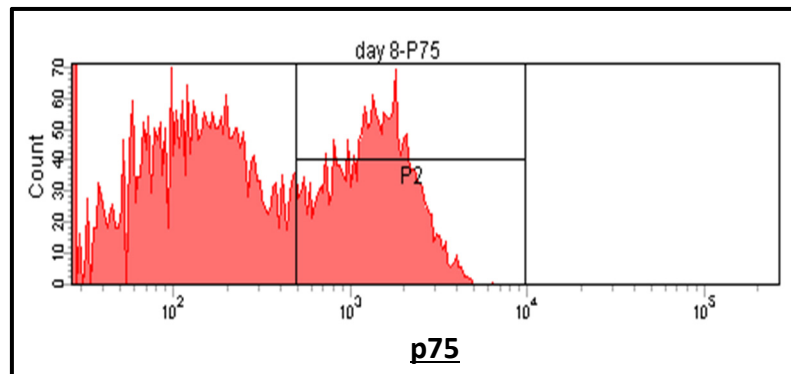

**B**

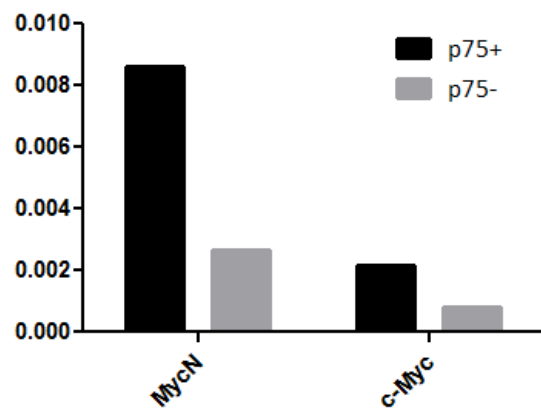

Supplement: S3 Fig — (A): FACS analysis shows the p75+ population for sorting. (B): The expression of MycN and c-Myc in p75+ and p75- populations determined by real-time PCR. (PDF) [file pone.0148062.s003.pdf]

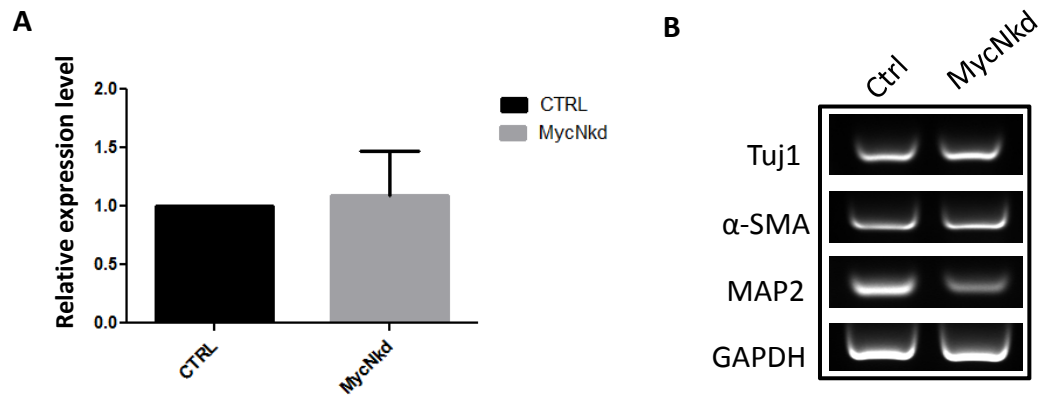

Supplement: S4 Fig — (A): Real time PCR analysis showed the knockdown of MycNdid not affect the expression of c-Mycin hNCSCs; (B): Control and MycNkd cells were plated on poly-D-lysine- and fibronectin-coated coverslips in differentiation media for 2 weeks and then determined the expression of SMA, Tuj1, and MAP2 by RT-PCR. (PDF) [file pone.0148062.s004.pdf]

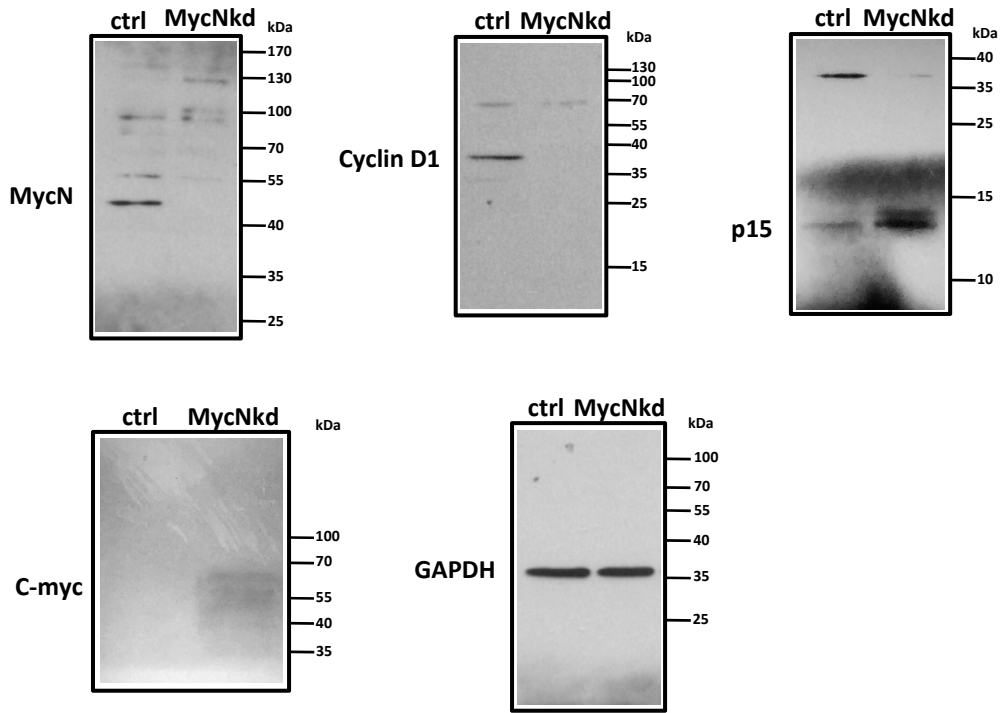

Supplement: S5 Fig — (PDF) [file pone.0148062.s005.pdf]
