# Supplementary material for: MycN Is Critical for the Maintenance of Human Embryonic Stem Cell-Derived Neural Crest Stem Cells
Source: PLoS One. 2016 Jan 27;11(1):e0148062. doi: 10.1371/journal.pone.0148062 (PMC4729679; doi:10.1371/journal.pone.0148062)
Supplement: S1 Table — (PDF) [file pone.0148062.s006.pdf]

*S1 Table Primer information of RT-PCR*

| Gene | | PRIMER SEQUENCE (5'-3') | Tm °C |
|---|---|---|---|
| **HNK-1** | forward | CTGGAGTCTGAGAAGGGGTC | 60 |
| | reverse | CGGAGACGCTCCGGACT | |
| **NTRK1** | forward | TCAACAAAATGTGGACGGAGA | 60 |
| | reverse | GTGGTGAACACAGGCATCAC | |
| **TH** | forward | CACGAAGTACTCCAGGTGGG | 60 |
| | reverse | CGAGCTGTGAAGGTGTTTGA | |
| **MYCN** 3 transcript variants | forward | CTTCGGTCCAGCTTTCTCAC | 60 |
| | reverse | GTCCGAGCGTGTTCAATTTT | |
| **MYCN** 2 transcript variants | forward | CACAGTGACCACGTCGATTT | 60 |
| | reverse | CACAAGGCCCTCAGTACCTC | |
| **SOX10** | forward | CTTTCTTGTGCTGCATACGG | 60 |
| | reverse | AGCTCAGCAAGACGCTGG | |
| **SNAIL** | forward | TTTACCTTCCAGCAGCCCTA | 60 |
| | reverse | CCCACTGTCCTCATCTGACA | |
| **C-MYC** | forward | GCGTCCTGGGAAGGGAGATCCGGAGC | 60 |
| | reverse | TTGAGGGGCATCGTCGCGGGAGGCTG | |
| **PHOX2α** | forward | CTTCCAATACAGCCCCCTG | 58 |
| | reverse | ATGGCTCTGGGAAGAACTTG | |
| **PHOX2β** | forward | CTTCGCGGAGACTCACTACC | 60 |
| | reverse | GTCGTCCCTGGAAGAGTCAG | |
| **CCND1** | forward | TCCTCTCCAAAATGCCAGAG | 60 |
| | reverse | GGCGGATTGGAAATGAACTT | |
| **BMI1** | forward | TTCATTGATGCCACAACCAT | 60 |
| | reverse | CATTTTTGAAAAGCCCTGGA | |
| **HAND2** | forward | CAGCAACGACAAGAAAACCA | 60 |
| | reverse | GGATGATTCCAAATGCAAGG | |
| **GATA3** | forward | CTCATTAAGCCCAAGCGAAG | 60 |
| | reverse | TTTTTCGGTTTCTGGTCTGG | |
| **EZH2** | forward | TTGTCAATGTAGTTCAGAG | 60 |
| | reverse | GTAGCACGGGCACTGCTTG | |
| **MASH1** | forward | CGGCCAACAAGAAGATGAGT | 60 |
| | reverse | CCGACGAGTAGGATGAGACC | |
| **Peripherin** | forward | AAGACGACTGTGCCTGAGGT | 60 |
| | reverse | ATGCAGAGGGAGAGGAGACA | |
| **NGFR** | forward | ACCTCATCCCTGTCTATTGCTCC | 60 |
| | reverse | GCTGTTGGCTCCTTGCTTGTT | |
| **CDKN2α** | forward | CTTCCTGGACACGCTGGT | 60 |
| | reverse | CATGGTTACTGCCTCTGGTG | |
| **CDKN2β** | forward | GGGGACTAGTGGAGAAGGTG | 60 |
| | reverse | GGTGAGAGTGGCAGGGTC | |
| **P21** | forward | CGACTGTGATGCGCTAATGG | 60 |
| | reverse | CTGCCTCCTCCCAACTCATC | |
| **Tuj1** | forward | CCAGCTGCAAGTCCGAGT | 58 |
| | reverse | CGCCCAGTATGAGGGAGAT | |
| **MAP2** | forward | GCCTTGTTATTTCTCCTGCAA | 60 |
| | reverse | GAGGAAGCATTGATTGGGAG | |
| **α-SMA** | forward | AGGAAGGACCTCTATGCTAACAAT | 58 |
| | reverse | AACACATAGGTAACGAGTCAGAGC | |
| **GAPDH** | forward | AGGGTCATCATCTCTGCC | 60 |
| | reverse | CCATCACGCCACAGTTTC | |
